# Clinical predictors of chronic rhinosinusitis: do the Canadian clinical practice guidelines for acute and chronic rhinosinusitis predict CT-confirmation of disease?

**DOI:** 10.1186/s40463-017-0243-x

**Published:** 2017-12-04

**Authors:** Paige Moore, Brian Blakley, Eric Meen

**Affiliations:** Department of Otolaryngology- Head & Neck Surgery, University of Manitoba, GB 421-820 Sherbrook Street, Winnipeg, MB R3A 1R9 Canada

**Keywords:** Chronic rhinosinusitis, Canadian guidelines, Predict

## Abstract

**Background:**

The diagnosis of chronic rhinosinusitis (CRS) based on clinical presentation alone remains challenging. To improve the accuracy of clinical diagnosis, the Canadian Rhinosinusitis Guidelines recommend the use of specific symptom and endoscopic criteria. Our study objective was to determine whether symptom and endoscopic criteria, as defined by the Canadian Rhinosinusitis Guidelines, accurately predict CT-confirmed CRS diagnosis.

**Methods:**

A retrospective cohort study of 126 patients who underwent CT sinuses based on clinical suspicion of possible CRS. The presence of symptom and endoscopic criteria, as defined by the Canadian Rhinosinusitis Guidelines, were compared between patients with and without a CT-confirmed CRS diagnosis using two-tailed Fisher’s exact tests. Positive predictive values and likelihood ratios were determined for each symptom and endoscopic finding.

**Results:**

Overall, 56.3% of patients had a CT-confirmed diagnosis of CRS. With the exception of nasal polyps, none of the symptom or endoscopic criteria had a statistically significant correlation with positive CT sinuses. For symptom criteria, positive predictive values ranged from 52.4% to 63.4%; likelihood ratios ranged from 0.85 to 1.34. For endoscopic criteria, positive predictive values and likelihood ratios were 71.4% and 1.94 (edema); 63.0% and 1.32 (discharge); and 92.9% and 10.1 (nasal polyps). 35.2% of patients with CT-confirmed CRS had normal endoscopic exams.

**Conclusion:**

The Canadian Rhinosinusitis Guidelines’ symptom and endoscopic criteria for CRS, with the exception of nasal polyps on endoscopy, do not accurately predict CT-confirmed disease. In addition, a normal endoscopic exam does not rule out CRS.

## Background

Chronic rhinosinusitis (CRS) is one of the most prevalent chronic diseases in the developed world [1]. The estimated prevalence of CRS in Canada is 5% [2], rivaling that of other common diseases such as diabetes mellitus and asthma [3]. The socioeconomic burden of CRS is profound, costing the US healthcare system over 60 Billion dollars per year [2], and lost productivity costs exceeding 13 Billion per year [4]. In addition, it has a significant impact on quality of life, comparable to CHF, angina, COPD, and back pain [1].

The diagnosis of CRS based on symptoms alone has been a longstanding challenge. Despite multiple revisions to diagnostic criteria over the past 20 years, the symptom-based diagnostic criteria still have a specificity of only 2–12%, and a positive predictive value (PPV) of 35–54% [5–7]. For this reason, the current international guidelines (International Consensus Statement on Allergy and Rhinology: Rhinosinusitis, 2016) recommend objective confirmation of disease using either nasal endoscopy or CT scan to increase the accuracy of diagnosis [8].

The Canadian Clinical Practice Guidelines for acute and chronic rhinosinusitis [9] define five major symptoms for diagnosing CRS: facial congestion/fullness; facial pain/pressure/fullness; nasal obstruction/blockage; purulent anterior/posterior nasal drainage (may be nondiscoloured or nonpurulent); and hyposmia/anosmia. In the Canadian Guidelines, these five cardinal symptoms are collectively given the acronym “CPODS”. At least 2 symptoms must be present for at least 8–12 weeks, as well as objective confirmation of disease on endoscopy or CT scan, for a diagnosis of CRS. Although the accuracy of the American CRS guidelines has been studied [6, 7, 10, 11], the accuracy of the Canadian guidelines has not. We therefore sought to determine whether symptom and endoscopic criteria, as defined by the Canadian Rhinosinusitis Guidelines, predict CT-confirmed CRS. We hypothesized that certain symptom criteria, specifically hyposmia and discharge, and endoscopic findings of purulence or polyps would be more strongly predictive of disease than other symptoms – as prior literature has demonstrated is the case for American CRS criteria [6–8, 10, 11].

## Methods

This was a retrospective cohort study. We identified all CT sinuses imaging studies ordered for evaluation of possible CRS through a tertiary level rhinology clinic between January 2014 and March 2016. To achieve this, we used a comprehensive imaging database to view all CT sinuses ordered in that time frame. Patients were excluded if there was evidence of previous endoscopic sinus surgery (ESS), sinonasal mass, symptom duration <8 weeks, age < 18 years, or existing diagnosis of CRS. A chart review was performed for each of these patients The following variables were extracted from routine questionnaires for past medical history: age, gender, smoking status (yes/no), history of asthma (yes/no), and history of nasal allergy (yes/no). Symptoms of CRS (congestion, facial pain/pressure/fullness, nasal obstruction, discolored nasal mucus, hyposmia/anosmia), and findings on nasal endoscopy (middle meatal edema, purulence, polyps) were documented during the patient interview and physical exam. Patients were considered symptom positive based on the Canadian guidelines if 2 or more of these symptoms were present for >8–12 weeks.

The CT studies were assigned a Lund-Mackay score [12], with a score of ≥4 considered diagnostic for CRS [13]. Of note, no patients demonstrated severe single-sinus or isolated disease in the absence of positive disease elsewhere (ie Lund-Mackay <4 despite positive CT findings). We then compared the symptom profiles and endoscopic findings of patients with and without CT-confirmed CRS (CT+ and CT-, respectively), determining sensitivity, specificity, PPV, NPV, and positive likelihood ratio (LR) for each symptom and endoscopic finding listed in Table 1. The statistical significance CT + versus CT - was assessed by a single Crosstabs analysis in IBM SPSS v24 (IBM Corporation, Armonk, NY) for dichotomous variables which defaults to Fischer’s exact test for small sample sizes (n less than25). For age, the continuous variable, the *t*-test was applied. The significance level was 0.05.

**Table 1:** Patient characteristics, symptoms, and endoscopic findings

	Non-CRS (CT-) *n* = 55	CRS (CT+) *n* = 71	p*	Sensitivity	Specificity	PPV	NPV	+LR
Age in years [mean (+/−SD)]	45 (14.7)	48(15.7)	0.249					
Sex								
Male, n (%)	18(33)	37(52)	0.047					
Female, n (%)	37(67)	34(48)						
Medical History								
History of asthma, n (%)	11(20)	7(10)	0.099	9.9	80	38.9	40.7	0.495
History of smoking, n (%)	4(7)	8(11)	0.250	11.3	92.7	66.7	44.7	1.54
History of allergies, n (%)	5(9)	8(11)	0.463	11.3	94.5	61.5	44.2	2.05
CPODS criteria								
Congestion, n (%)	24 (44)	37(52)	0.373	52.1	56.4	60.7	47.7	1.19
Pain/pressure/fullness, n (%)	40 (73)	44(62)	0.254	62.0	27.3	52.4	35.7	0.85
Obstruction, n (%)	30 (55)	45(63)	0.362	63.4	45.5	60.0	49.0	1.16
Discharge, n (%)	31 (56)	52(73)	0.059	73.2	43.6	62.7	55.8	1.30
Smell loss, n (%)	15 (27)	26(37)	0.338	36.6	72.7	63.4	47.1	1.34
CPODS + (at least 2/5), n (%)	46 (84)	62(87)	0.795	87.3	16.4	57.4	50.0	1.04
Endoscopic findings								
Purulence, n (%)	4 (7)	10 (14)	0.226	14.1	92.7	71.4	45.5	1.94
Polyps, n (%)	1 (2)	13 (18)	**0.003**	18.3	98.2	92.9	48.2	10.07
Middle meatal edema, n (%)	20 (36)	34 (48)	0.209	47.9	63.6	63.0	48.6	1.32

*2-sided Fischer’s exact test. Significant values in bold

## Results

A total of 190 CT sinuses were identified. After reviewing CT scans and charts, the following patients were excluded: existing diagnosis of CRS (*n* = 3); nasal mass (*n* = 10); previous endoscopic sinus surgery (*n* = 47); and duration <12 weeks or diagnosis of recurrent acute bacterial sinusitis (*n* = 4); resulting in 126 patients included. 86% of patients met the Canadian symptom criteria for CRS. Overall, 56.3% of patients had CT-confirmed diagnosis of CRS.

Table 1 shows sensitivity, specificity, positive predictive value (PPV), negative predictive value (NPV), *p* values, and likelihood ratios for all variables**.** Sensitivity of the individual symptoms ranged from 36.6–73.2%, the most sensitive symptom being nasal discharge. Specificity of individual symptoms ranged from 27.3–72.7% with the most specific being smell loss. None of the individual symptoms were strongly predictive of disease (PPV 52.4–63.4%), nor did they demonstrate a statistically significant difference between groups with and without CRS. The Canadian symptom criteria collectively (i.e. two or more CPODS symptoms) showed high sensitivity (87.3%) and low specificity (16.4%), but did not show a statistically significant difference between the CT+ and CT- groups.

Endoscopic evidence of polyps was highly specific and predictive of disease, and showed a statistically significant difference between CT+ and CT- groups (specificity =98.2%; PPV =92.9%, *p* = 0.003, LR = 10.07). Findings of discharge or middle meatal edema were less strongly predictive (PPV = 71.4 and 63.0, respectively) and showed no statistically significant difference between groups. A substantial number of patients with CRS had a normal endoscopic exam (35.2%).

## Discussion

Our results demonstrate that The Canadian CRS symptom criteria have poor specificity and PPV (16.4% and 57.4%, respectively), comparable to other international CRS guidelines (specificity = 2–12%; PPV = 35–54%) [5–7]. Unlike studies of the American criteria [6–8, 10, 11], we found that no single symptom has significant difference between groups with and without CRS.

Endoscopic examination is a routine and important step in evaluating patients for CRS. The presence of nasal polyps was highly predictive of disease (PPV 92.9%), and was the only variable in our study that demonstrated statistically significant difference between CT+ and CT- groups (*p* = 0.02). Conversely, edema and discharge were relatively non-predictive, and were not significantly different between groups. We found that 35.2% of patients with CRS have no endoscopic findings at all. This finding supports the current International Consensus Statement [8] that patients meeting symptom criteria with a normal endoscopy should have a CT scan. It also demonstrates that a normal endoscopy does not rule out disease – with approximately one third of CRS patients requiring CT scan for diagnosis. The utility of CT scans in diagnosing CRS remains challenging. While it holds a well-recognized role in patients with high clinical suspicion and negative endoscopy [8], it is crucial that it not be adopted as a screening tool for every patient presenting with sinonasal symptoms; but be used judiciously in cases where there is sufficient suspicion of CRS and the absence of other sinonasal conditions that entirely explain the patient’s clinical presentation. Given the markedly heterogeneous nature of CRS, the physician must consider symptomatology and physical exam findings- as well as imaging studies when required -to arrive at a diagnosis.

The primary limitation of our study is potential selection bias due to our focus on patients for whom we had a high index of suspicion for CRS. However, because we evaluated the guidelines in patients for whom clinical suspicion was high enough to proceed with imaging, this cohort is representative of patients presenting with possible CRS, and therefore is applicable to any otolaryngology practice in which patients are routinely undergoing assessment for possible inflammatory sinus disease. In addition, there may be reporting bias present for variables such as smoking status, allergies, and asthma. These dichotomous (yes/no) variables were extracted by patient-filled questionnaire, and were not formally assessed with pulmonary function tests, skin prick testing, or other objective measures. We therefore did not perform in-depth analyses of these variables as they pertain to CRS. The designation of Lund-Mckay ≥4 as diagnostic for CRS also presents some limitations. Firstly, although this cutoff is widely accepted in rhinologic literature, it does not account for patients with marked single-sinus or unilateral disease. Second, we have no way of documenting symptomatology at the time of CT scan. The extent of disease could be over- or under-reported, depending on the patient’s condition at the time of scan (ie acute viral illness, or dramatic response to medical therapy). Similarly, their condition may have improved by the time of their CT scan if treatment was initiated at their first visit. However, we feel that the cohort of patients converting from a positive to a negative scan (ie Lund-Mackay <4) after therapy initiation (such as systemic steroids or more commonly, intranasal corticosteroids) would be exceedingly small. This could be addressed in future prospective studies by withholding treatment until baseline imaging is obtained.

CRS continues to present a diagnostic challenge. Although certain symptoms (such as hyposmia and purulent rhinorrhea) have been ascribed high predictive values in the literature [7, 10], this was not reproduced in our study. The 2016 International Consensus Statement on Allergy and Rhinology: Rhinosinusitis [8] recommends against the diagnosis of CRS based on symptoms alone due to the poor specificity and PPV, which is reflected in the Canadian CRS criteria. Despite numerous international revisions in recent years, symptom criteria for CRS continue to have low specificity and modest PPV. Additionally, in a sizeable subset of patients with CRS (approximately one third), endoscopy was normal- underscoring the importance of imaging of patients in whom the endoscopic exam is negative but the history is suggestive of CRS.

## Conclusion

The Canadian Clinical Practice Guidelines for acute and chronic rhinosinusitis define symptom and endoscopic criteria for CRS, which apart from nasal polyps on endoscopy, are not highly predictive of CT-confirmed disease. In addition, negative endoscopy does not reliably rule out CRS. These results highlight the ongoing diagnostic challenge presented by CRS.

## Abbreviations

CHF: Congestive heart failure
COPD: Chronic obstructive pulmonary disease
CPODS: Canadian CRS guildeline clinical criteria: congestion; pain/pressure; obstruction; discharge; smell loss
CRS: Chronic rhinosinusitis
CT/CT+/CT-: Computed tomography; positive scan; negative scan
LR: Likelihood ratio
NPV: Negative predictive value
PPV: Positive predictive value
US: United States

## References

[1] Halawi AM, Smith SS, Chandra RK (2013). Chronic rhinosinusitis: epidemiology and cost. Allergy Asthma Proc.

[2] Caulley L, Thavorn K, Rudmik L, Cameron C, Kilty SJ. Direct costs of adult chronic rhinosinusitis by using 4 methods of estimation: results of the US medical expenditure panel survey. In: J Allergy Clin Immunol. Vol 136. Mosby; 2015:1517–1522. doi:10.1016/j.jaci.2015.08.037. 10.1016/j.jaci.2015.08.037 26483176

[3] Rudmik L. Chronic rhinosinusitis: an under-researched epidemic. J Otolaryngol Head Neck Surg. 2015;44(11) doi:10.1186/s40463-015-0064-8. 10.1186/s40463-015-0064-8 PMC4377210 25890357

[4] Rudmik L, Soler ZM, Smith TL, Mace JC, Schlosser RJ, DeConde AS (2015). Effect of continued medical therapy on productivity costs for refractory chronic rhinosinusitis. JAMA Otolaryngol Neck Surg.

[5] Fokkens WJ, Lund VJ, Mullol J (2012). European position paper on rhinosinusitis and nasal polyps.

[6] Bhattacharyya N, Lee LN (2010). Evaluating the diagnosis of chronic rhinosinusitis based on clinical guidelines and endoscopy. Otolaryngol Neck Surg.

[7] Pynnonen M, Fowler K, Terrell JE (2007). Clinical predictors of chronic rhinosinusitis. Am J Rhinol.

[8] Orlandi RR, Kingdom TT, Hwang PH (2016). International consensus statement on allergy and rhinology: rhinosinusitis. Int Forum Allergy Rhinol..

[9] Desrosiers M, Evans GA, Keith PK (2011). Canadian clinical practice guidelines for acute and chronic rhinosinusitis. J Otolaryngol Head Neck Surg.

[10] Hsueh WD, Conley DB, Kim H (2013). Identifying clinical symptoms for improving the symptomatic diagnosis of chronic rhinosinusitis. Int Forum Allergy Rhinol.

[11] Bhattacharyya N. Clinical and symptom criteria for the accurate diagnosis of chronic rhinosinusitis. Laryngoscope. 2006:116(S110):1–22. doi:10.1097/01.mlg.0000224508.59725.19. 10.1097/01.mlg.0000224508.59725.19 16826087

[12] Lund VJ, Kennedy DW. Staging for rhinosinusitis http://journals.sagepub.com.uml.idm.oclc.org/doi/pdf/10.1016/S0194-59989770005-6. Accessed March 7, 2017.

[13] Bhattacharyya N, Fried MP (2003). The accuracy of computed tomography in the diagnosis of chronic rhinosinusitis. Laryngoscope.

